# Identification of a substrate-like cleavage-resistant thrombin inhibitor from the saliva of the flea *Xenopsylla cheopis*

**DOI:** 10.1016/j.jbc.2021.101322

**Published:** 2021-10-21

**Authors:** Stephen Lu, Lucas Tirloni, Markus Berger Oliveira, Christopher F. Bosio, Glenn A. Nardone, Yixiang Zhang, B. Joseph Hinnebusch, José M. Ribeiro, John F. Andersen

**Affiliations:** 1 Laboratory of Malaria and Vector Research, National Institute of Allergy and Infectious Diseases, Bethesda, Maryland, USA; 2 Laboratory of Bacteriology, National Institute of Allergy and Infectious Diseases, Hamilton, Montana, USA; 3 Centro de Pesquisa Experimental, Hospital de Clínicas de Porto Alegre, Porto Alegre, Rio Grande do Sul, Brazil; 4 Research Technologies Branch, National Institute of Allergy and Infectious Diseases, Bethesda, Maryland, USA

**Keywords:** blood feeding, X-ray crystallography, flea, thrombin, aPTT, activated partial thromboplastin time, PAR-1, protease-activated receptor-1, PBS, phosphate-buffered saline, PT, prothrombin time, SGH, salivary gland homogenate, TFA, trifluoroacetic acid, TT, thrombin time, TTI, tsetse thrombin inhibitor

## Abstract

The salivary glands of the flea *Xenopsylla cheopis*, a vector of the plague bacterium, *Yersinia pestis*, express proteins and peptides thought to target the hemostatic and inflammatory systems of its mammalian hosts. Past transcriptomic analyses of salivary gland tissue revealed the presence of two similar peptides (XC-42 and XC-43) having no extensive similarities to any other deposited sequences. Here we show that these peptides specifically inhibit coagulation of plasma and the amidolytic activity of α-thrombin. XC-43, the smaller of the two peptides, is a fast, tight-binding inhibitor of thrombin with a dissociation constant of less than 10 pM. XC-42 exhibits similar selectivity as well as kinetic and binding properties. The crystal structure of XC-43 in complex with thrombin shows that despite its substrate-like binding mode, XC-43 is not detectably cleaved by thrombin and that it interacts with the thrombin surface from the enzyme catalytic site through the fibrinogen-binding exosite I. The low rate of hydrolysis was verified in solution experiments with XC-43, which show the substrate to be largely intact after 2 h of incubation with thrombin at 37 °C. The low rate of XC-43 cleavage by thrombin may be attributable to specific changes in the catalytic triad observable in the crystal structure of the complex or to extensive interactions in the prime sites that may stabilize the binding of cleavage products. Based on the increased arterial occlusion time, tail bleeding time, and blood coagulation parameters in rat models of thrombosis XC-43 could be valuable as an anticoagulant.

Thrombin is a serine protease that plays a central role in hemostasis, catalyzing the conversion of fibrinogen to fibrin, activating platelets through cleavage of protease-activated receptor-1 (PAR-1) and potentiating its own production through activation of factors V, VIII, and XI (1). The thrombin catalytic site is composed of the triad His^57^, Asp^102^, and Ser^195^ lying at the bottom of a cleft formed by two extensions in the basic trypsin-like structure known as the 60 loop and the autolysis loop. The structure of this cleft limits the substrate specificity of thrombin to fibrinogen and those additional proteins involved with blood clotting and inflammation listed above. Thrombin also possesses two positively charged binding sites at other points on its surface, known as the fibrinogen-binding site (exosite I) and the heparin-binding site (exosite II). These provide additional points of interaction for the substrate that serve to orient it and enhance its binding affinity as well as providing an interaction site for charged glycans (2). Naturally occurring thrombin inhibitors have been isolated from numerous blood-feeding animals, which utilize the catalytic and exosites of thrombin in a variety of ways to attain very high binding affinities (3, 4).

Over the last 20 years, fueled by advances in sequencing technology, many pharmacologically active proteins and peptides from the salivary glands of blood-feeding arthropods including mosquitoes (5), flies (6), kissing bugs (7), ticks (8) and fleas (9) have been identified, leading to the discovery of numerous molecules that potently modulate hemostasis in vertebrate hosts. One group of blood feeders, the fleas, belong to the insect order Siphonaptera, which contains over 2500 known species organized into 238 genera (10). From the medical point of view, they are relevant for both human and veterinary health, acting as vectors for several pathogens including *Bartonella* sp., *Rickettsia* sp., and *Yersinia pestis*, the etiological agent of bubonic plague (11). Despite their importance, few flea salivary proteins have been functionally characterized, although many novel sequences have been identified in the previously published salivary transcriptomes of the rat flea *Xenopsylla cheopis* (12) and the cat flea *Ctenocephalides felis* (9). In this study we investigate the activity of two similar, highly expressed peptides given the names XC-42 (ABM55431.1) and XC-43 (ABM55432.1) that have no obviously conserved domains or overall similarities with other peptides of known function (12). We show these peptides to be present in the flea salivary gland, to tightly bind thrombin and to inhibit its proteolytic activity. We have determined the crystal structure of the XC-43-thrombin complex obtained at a resolution of 2.15 Å, and it reveals a substrate-like binding mode for the peptide but an unexpected absence of proteolytic cleavage. As suggested by the presence of a short sequence motif reminiscent of those found in a number of thrombin exosite I-binding peptides, XC-43 also interacts intimately with the fibrinogen-binding exosite. Finally, using an animal model we show that XC-43 inhibits blood coagulation pathways *in vitro* and *in vivo*. The study describes a novel peptide of nearly minimal size for function in an active site/exosite I-binding mechanism that is also resistant to cleavage by the protease. This combination of features results in very high-affinity binding and a high degree of selectivity for thrombin using standard L-amino acids without any apparent posttranslational modification of the peptide.

## Results

### *X. cheopis* saliva contains two specific inhibitors of thrombin

**Figure 1:**
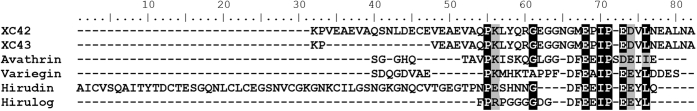
**Amino acid sequence alignment of mature XC-42 (ABM55431.1), XC-43 (ABM55432.1), variegin (P85800.1), avathrin (5GIM_D), hirudin (P09945.1), and hirulog 1 (hirulog is a synthetic sequence, but is added to the alignment for comparison).** Identical residues are *black boxed* while similar residues are *gray boxed*.

The salivary gland transcriptome, or sialome, of the rat flea *X. cheopis* (12) contained two similar peptides (78% of identity), given the names XC-42 (ABM55431.1) and XC-43 (ABM55432.1), with no known conserved domains or overall similarities to other deposited proteins. The two peptides are nearly identical in sequence, but in XC-42 a duplication and insertion result in the peptide being 14 amino acids longer than XC-43. When aligned with thrombin inhibitors isolated from other blood feeding animals, including variegin and avathrin from ticks, as well as hirudin from the medicinal leech *Hirudo medicinalis*, we observed the presence of a conserved motif E-x-I-P-x(0,1)-[ED]-x-[L] (in “PROSITE” notation, 0,1 indicates that x represents 0 or 1 residue) (13) near the C-terminus of the peptide (Fig. 1). In naturally occurring thrombin inhibitory peptides, this motif is associated with binding to the anionic fibrinogen-binding exosite (exosite I), suggesting that XC-42 and XC-43 may inhibit thrombin. Also, located 13 residues N-terminal to the putative exosite-I-binding motif is the dipeptide Pro 10-Lys 11, which could serve as a P2-P1 sequence interacting with the catalytic site region. The putative exosite I-binding region, catalytic site-binding region, and the length of the spacer region between them are very similar to the tick peptides variegin and avathrin and suggested strongly that the flea peptides are also catalytic site/exosite-I binding inhibitors of thrombin (Fig. 1). Both XC-42 and XC-43 possess a putative signal peptide predicted to be cleaved between residues 23 and 24, rendering mature peptides of 5.38 kDa and pI of 3.95 for XC-42 and 3.86 kDa and pI of 4.31 for XC-43. Mass spectral analysis of *X. cheopis* salivary gland homogenates (SGH) shows the presence of unique fragment peptides derived from both molecules (Fig. S1 and Table S1), confirming that XC-42 and XC-43 are present in the gland. Unmodified peptides covering nearly the entire sequence were identified, suggesting that posttranslational modifications affecting inhibitory activity (14) may not be present.

**Figure 2:**
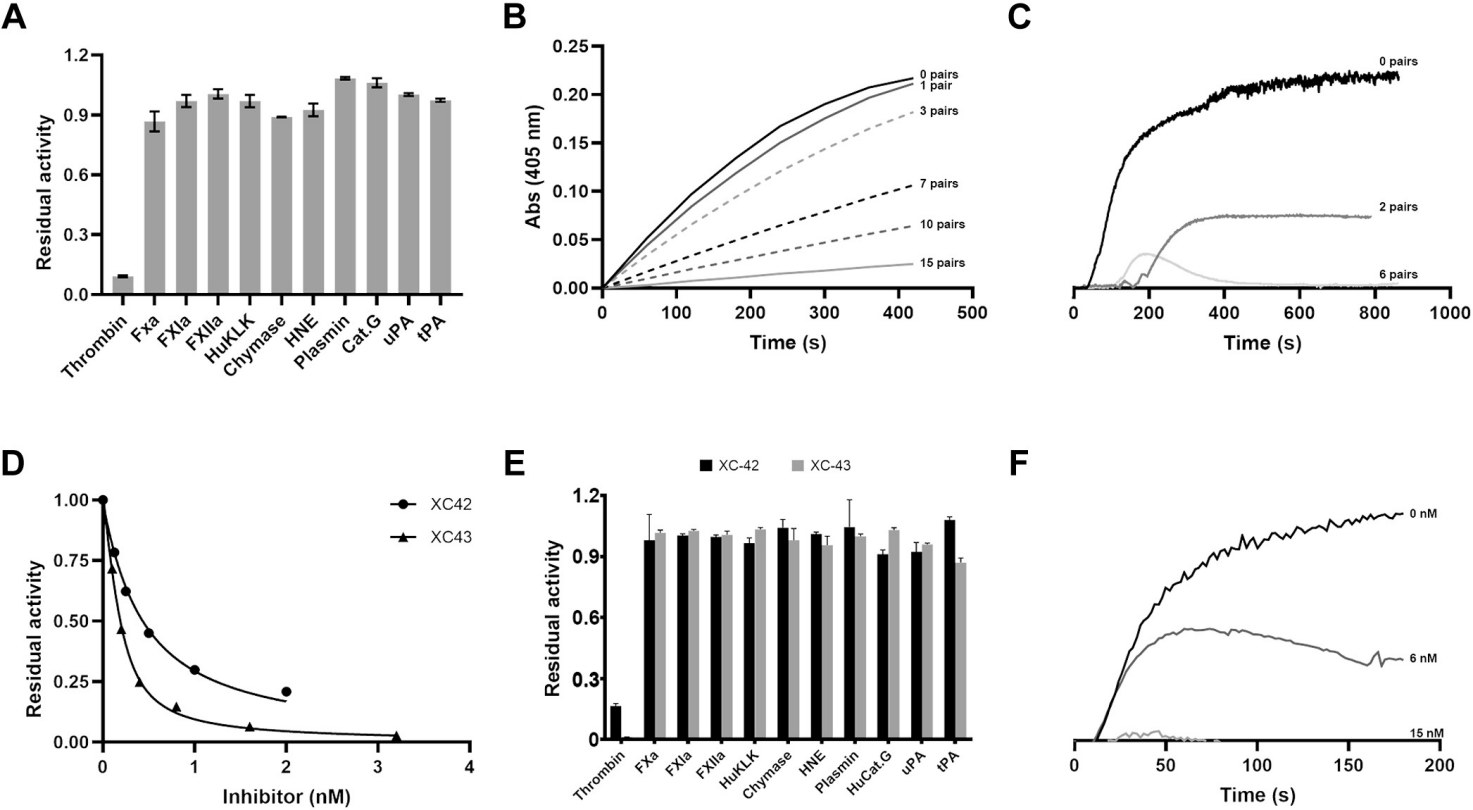
**Inhibition of coagulation of plasma and the activity of serine proteases by salivary gland homogenate, XC-42 and XC-43.***A*, the activity of different serine peptidases was measured in the presence of *X. cheopis* SGH (1 μg) relative to the presence of buffer alone. *B*, progress curves of thrombin-catalyzed hydrolysis of S-2238 in the presence of increasing quantities of *X. cheopis* SGH. The number of gland pair equivalents of SGH represented by each trace in shown on the graph. *C*, effect of *X. cheopis* SGH on thrombin-induced platelet aggregation. Platelet aggregometer traces showing inhibition of aggregation of washed platelets in the presence of increasing quantities of *X. cheopis* SGH. The number of gland pair equivalents of SGH represented by each trace is shown on the graph. The thrombin concentration was 3 nM. *D*, concentration dependence of thrombin inhibition by XC-42 and XC-43 using S-2238 as substrate at a concentration of 100 μM. Fitting of the data to the Morrison equation (for tight binding inhibitors) produces *Ki* values of 1.8 × 10^−11^ M for XC-42 and 6.1 × 10^−12^ M for XC-43. *E*, XC-42 and XC-43 inhibitory activity toward a panel of serine proteases including several from the coagulation cascade using chromogenic substrates. Abbreviations are the same as in panel *A*. *F*, concentration dependence of XC-43-mediated inhibition of thrombin-induced platelet aggregation. CatG, cathepsin G; FXa, factor Xa; FXIa, factor XIa; FXII, factor XIIa; HNE, neutrophil elastase; HuKLK, human kallikrein; tPA, tissue plasminogen activator; uPA, urokinase plasminogen activator.

*X. cheopis* SGH was tested for its ability to inhibit serine proteases involved in hemostasis and inflammation using small peptidomimetic substrates. The panel included proteases from the coagulation cascade, as well as the fibrinolytic and inflammatory pathways. Of this group, only thrombin was significantly inhibited (Fig. 2*A*). SGH also produced a concentration-dependent inhibition of hydrolysis of the chromogenic thrombin substrate S-2238 (Fig. 2*B*) and inhibited thrombin-mediated activation of PAR-1 in washed platelet preparations (Fig. 2*C*), strongly suggesting that the extract contains a specific thrombin inhibitor. We found that synthetic XC-42 and XC-43 both inhibit cleavage of S-2238 by purified α-thrombin in a concentration-dependent manner (Fig. 2*D*). Additionally, we found that when the same panel of proteases tested with SGH was tested against XC-42 and XC-43, only thrombin was significantly inhibited (Fig. 2*E*) and that XC-43 potently inhibited thrombin-mediated activation of PAR-1 in washed platelets (Fig. 2*F*). Together these data indicate that XC-42 and XC-43 are largely responsible for the inhibition of thrombin seen with crude SGH, suggesting that they may be the primary inhibitors of the coagulation cascade utilized during blood feeding by *X. cheopis*.

**Figure 3:**
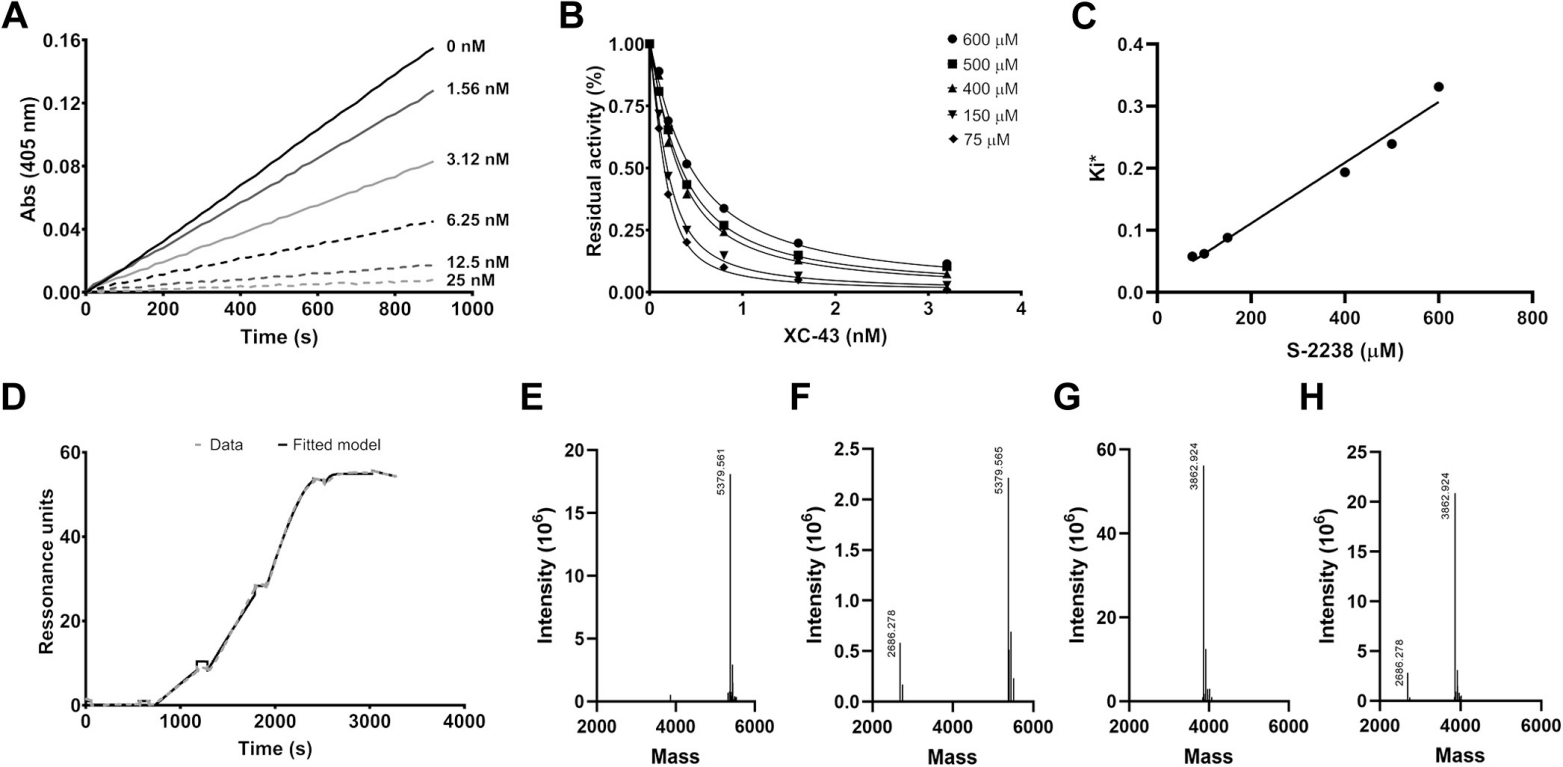
**XC-43 is a fast, competitive tight-binding thrombin inhibitor.***A*, progress curves of thrombin-catalyzed hydrolysis of S-2238 in the presence of different concentrations of XC-43. *B*, analysis of S-2238 hydrolysis by thrombin in the presence of increasing concentrations of XC-43 by fitting to the Morrison equation for tight-binding inhibitors. *C*, linear regression of the apparent *K*_i_ (*K*_i_^app^) *versus* S-2238 concentration indicates that XC-43 is a competitive tight-binding thrombin inhibitor. *D*, surface plasmon resonance in the single cycle mode of thrombin binding to biotinylated XC-43 bound to neutravidin that is immobilized on the chip surface. Analyte concentrations were: 0 pM, 93.75 pM, 187.5 pM, 375 pM, 750 pM. The K_D_ was determined by fitting experimental data to a 1:1 binding model (*dashed line*). *E*–*H*, cleavage of XC-42 and XC-43 by thrombin as measured in solution by mass spectrometry at 25:1 inhibitor:thrombin molar ratios. *E*, XC-42 control without thrombin, (*F*) 25 μM XC-42 with 1 μM thrombin, (*G*) XC-43 control without thrombin, (*H*) 25 μM XC-43 with 1 μM thrombin incubated for 2 h at 37 °C. Mass values of the graph correspond to the mass of intact XC-42 (5379 Da), XC-43 (3862 Da) and the cleavage fragment LYQRGEGGNGMEPIPEDVLNEALNA (2686 Da).

Progress curves observed after initiation of the proteolytic reaction by addition of thrombin to a mixture containing increasing concentrations of XC-43 show the peptide to be a fast-binding inhibitor (Fig. 3*A*). The substrate (S-2238) concentration dependence of inhibition was consistent with a competitive mechanism exhibiting an inhibitory constant (*K*_*i*_) of 7.7 × 10^−12^ M (Fig. 3, *B* and *C*). The *Ki* for inhibition of thrombin by XC-42 calculated from the data presented in Figure 2*D* was 18 × 10^−12^ M indicating that the two peptides inhibit thrombin with similar potency. Moreover, measurement of thrombin binding to a biotinylated XC-43-bound plasmon resonance surface (SPR) produced a dissociation constant (K_D_) of 3.0 × 10^−12^ M with an association rate constant (ka) of 4.0 × 10^7^ M^−1^s^−1^ and a dissociation rate constant (kd) of 1.2 × 10^−4^ s^−1^ (Fig. 3*D*). Notably, SPR assays in which XC-42 and XC-43 were passed over a surface of immobilized thrombin exhibited elevated dissociation constants, indicating possible steric hindrance of inhibitor interaction, as has been seen for the catalytic site/exosite-I-binding inhibitor anophelin (15). Nevertheless, XC-42 and XC-43 produced very similar values for both the kinetic parameters and the dissociation constant, further indicating that the two inhibitors bind thrombin in a similar manner (Fig. S2). The tight binding of XC-43 with thrombin was also affirmed using ITC as being enthalpy-driven (Δ*H* = −29.5 ± 0.3 kcal/mol) (Fig. S3).

Since XC-42 and XC-43 act as competitive thrombin inhibitors, they may be susceptible to thrombin-mediated proteolysis as has been demonstrated for other substrate-like inhibitors (16, 17, 18, 19). When both inhibitors were incubated with thrombin for 2 h at 37 °C at a molar ratio of 25:1 (inhibitor:thrombin) and analyzed by mass spectrometry, major ions corresponding to the intact inhibitors, 5379 Da for XC-42 and 3862 Da for XC-43, were observed, and much smaller peaks corresponding in mass to the longer cleavage fragment Leu^12^–Ala^36^ (numbering refers to XC-43, 2886 Da) (Fig. 3, *E*–*H*) were also present, indicating that the majority of the peptide was uncleaved. XC-43 appeared especially resistant to cleavage, with the fragment ion intensity being less than 15 percent of that of the uncleaved peptide.

### The crystal structure of XC-43 in complex with thrombin

**Figure 4:**
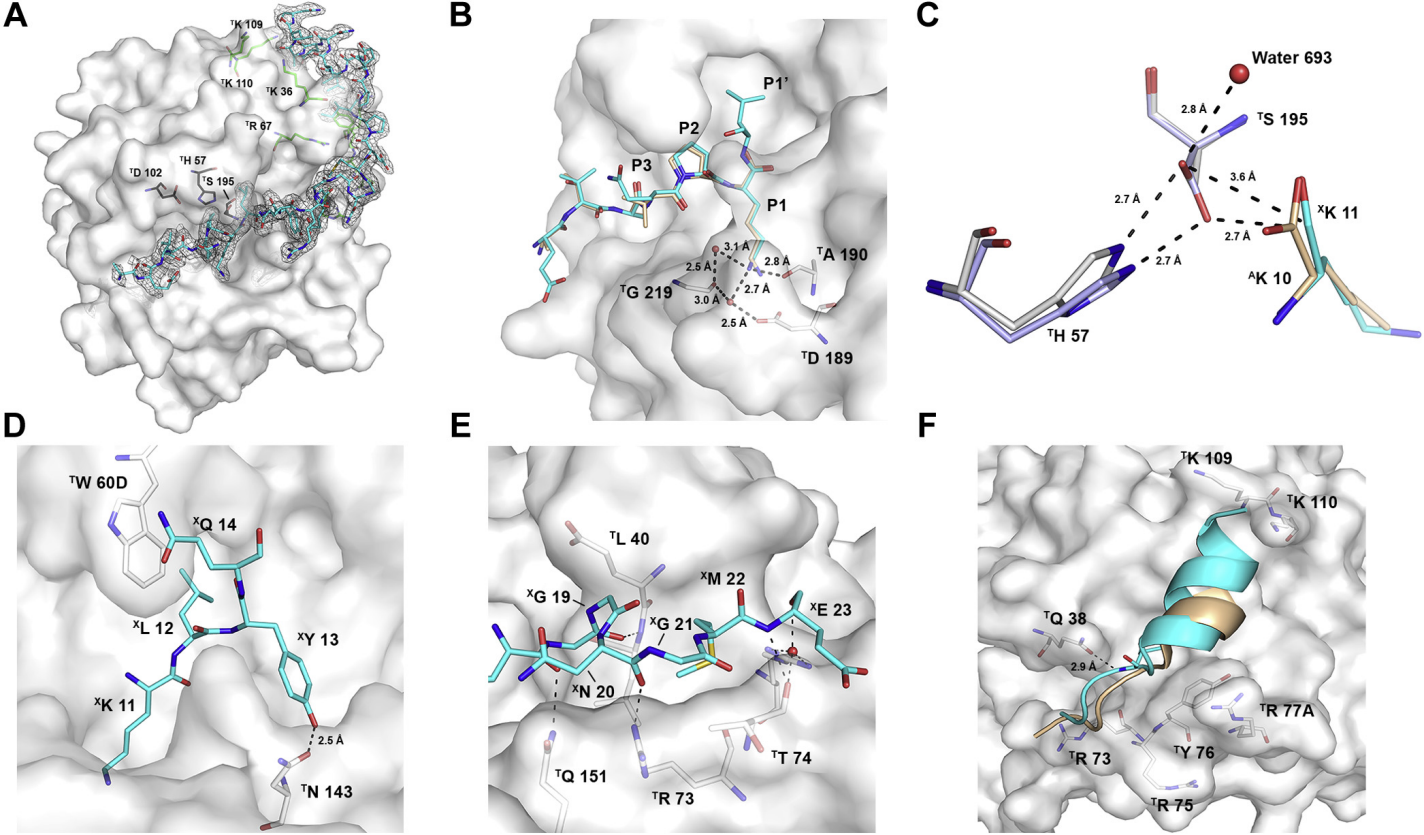
**Crystal structure of XC-43 complexed with human thrombin.***A*, complex of XC-43 (carbon atoms in *cyan*) with thrombin showing 2Fo – Fc electron density contoured at 1.0σ, covering bound XC-43 in the range from Glu 6 to Asn 35. Residues from the catalytic triad are shown in *stick* representation (carbon = *black*, oxygen = *red*, nitrogen = *blue*). Residues that compose the fibrinogen-binding site are also shown (carbon = *green*, oxygen = *red*, nitrogen = *blue*). *B*, interaction of the N-terminal regions of XC-43 (carbon = *cyan*, oxygen = *red*, nitrogen = *blue*) and avathrin (PDB accession number 5GIM (19), carbon = *beige*) with the thrombin surface. ^X^Lys 11 in the P1 position of XC-43 forms a hydrogen bond interaction with ^T^Asp 189 *via* an intervening water molecule. *C*, interactions of the P1 residue of XC-43 and avathrin with ^T^Ser 195 of thrombin. A buried water molecule at the XC-43-thrombin interface hydrogen bonds with the hydroxyl group of ^T^Ser 195, which is rotated 180° from its position in avathrin. Carbon atoms from thrombin complexed with XC-43 are represented in *light gray*, and thrombin residues from the avathrin complex are represented in *light blue*. The XC-43 P1 residue carbons are represented in *cyan* while avathrin P1 residue is represented in *beige*. Oxygen and nitrogen are shown in *red* and *blue*, respectively. Hydrogen bonds are shown as *black dashed lines* with distances (angstroms) shown. *D*, XC-43 P1ʹ–P3ʹ interactions with the thrombin prime sites. Thrombin is shown as a *gray surface* with some residues shown as *sticks*. Thrombin carbon atoms are shown in *light gray*, and XC-43 in *cyan*. Oxygen atoms are shown in *red* and nitrogen in *blue*. *E*, hydrogen bonds (shown as *black dashed lines* with distances in angstroms) formed between XC-43 residues ^X^Glu 17 to ^X^Glu 23 and thrombin. *F*, comparison between XC-43 (*cyan*) and avathrin (*beige*) positioning at the thrombin exosite I. Residues that are part of exosite I are shown as *sticks*. Distances shown are based on chains F and G from thrombin and chain P from XC-43. Nitrogen atoms are shown in *blue* while oxygen atoms are shown in *red*. *Blacked dashed line* represents the distance (Å) of between the indicated atoms.

The three-dimensional structure of human α-thrombin complexed with XC-43 was determined by X-ray crystallography at a resolution of 2.15 Å using molecular replacement methods with a thrombin search model (Table 1). The crystal belonged to the orthorhombic space group P2_1_2_1_2_1_ with an asymmetric unit containing six thrombin-XC-43 complexes. All six complexes have a similar overall structure (r.m.s.d. = 0.153 ± 0.0085 Å over 260 Cα positions) with high-quality electron density covering the peptide spanning from XC-43 residues ^X^Glu 6 (X superscript indicates XC-43 while T superscript indicates thrombin) to ^X^Asn 35 (Figs. 4*A*, S4 and S5). XC-43 binding buries an interface measuring 1710.5 Å^2^ (48% of the inhibitor surface area) and as suggested by the alignment described in Figure 1, binds with contact points at the protease active site and at exosite I (Fig. 4*A*). Moreover, binding of XC-43 does not cause major rearrangements in the thrombin backbone structure when the complex is compared with the structure of free thrombin (PDB 3U69 r.m.s.d. = 0.921 Å over 257 Cα positions (20) from the thrombin heavy chain).

**Table 1:** Data collection and refinement statistics for the XC-43-thrombin complex

Crystal	Thrombin/XC-43
Resolution (Å)	50–2.15
Beamline	22-ID
Wavelength (Å)	1.000
Completeness (total/high resolution shell)	99.9/100
Average Redundancy (total/high resolution shell)	11.2/11.6
R_merge_ (total/high resolution shell, %)	8.5/69.7
CC_1/2_ (total/high resolution shell)	99.9/94.2
I/sigI (total/high resolution shell)	18.3/4.2
Observed reflections	1,825,903
Unique Reflections	163,186
Space group	P2_1_2_1_2_1_
Unit cell dimensions (Å)	
a	113.3
b	136.3
c	193.9
α, β, γ (˚)	90
Refinement	
Total non-H protein atoms	15,074
Total non-H solvent atoms	1163
RMS deviations	
Bond lengths (Å)	0.007
Bond angles (˚)	0.868
Mean B factors (Å^2^)	
Protein	42.97
Solvent	45.43
Molprobity analysis	
Ramachandran plot (favored/allowed, %)	97.42/2.58
Clashscore	1.68
Rotamer outliers (%)	0.5
Coordinate error ML (Å, Phenix)	0.18
R_cryst/_R_free_	0.166/0.193

The N-terminal portion of the peptide follows a path in the substrate-binding groove that is almost identical to that of avathrin, an inhibitor from the tick *Amblyomma variegatum* (PDB 5GIM, Fig. 4*B*). In the active site region, the side chain of ^X^Lys 11 (P1) is inserted into the S1 pocket and its NZ atom is hydrogen bonded with the side chain of ^T^Asp 189 and the carbonyl oxygen of ^T^Gly219 through a network involving two water molecules. Also present is a hydrogen bond between the carbonyl oxygen of ^T^Ala 190 and NZ of ^X^Lys 11. Though the peptide shows a substrate-like binding conformation, no evidence of peptide bond cleavage is apparent (Fig. 4*B*). Because of this, the structure provides an unusually complete view of the interactions of a substrate-like molecule at subsites covering the active site region. The backbone atoms of ^X^Lys 11 are very similarly placed to those of other inhibitors such as avathrin and hirulog-1, although its carbonyl carbon is 0.5 to 0.9 Å further from Cα of ^T^Ser 195 than in these two inhibitors, suggesting that the scissile bond is positioned similarly in these complexes. The side chain of ^X^Leu 12 (P1ʹ) is situated in the pocket (S1ʹ) bounded by ^T^His 57, ^T^Trp 60D, ^T^Lys 60F, ^T^Leu 41, and the ^T^Cys 42-Cys59 disulfide bond with the side chain of Lys 60F being pushed toward ^T^Phe 60H by the bulky hydrophobic side chain of ^X^Leu 12. This positions a water molecule (^T^Wat 693) in the vicinity of the P1-P1ʹ peptide bond where it forms a hydrogen bond with the side chain hydroxyl of ^T^Ser 195 (Fig. S6). The serine side chain is rotated approximately 180° relative to its position in most other thrombin/inhibitor complex structures and its hydroxyl group continues to lie within hydrogen bonding distance of NE2 in ^T^His 57 and also forms a hydrogen bond with the carbonyl oxygen atom of ^X^Lys 11 of the inhibitor (3.07 Å, Fig. 4*C*). The repositioned ^T^Ser 195 hydroxyl lies 3.6 Å away from the carbonyl carbon of ^X^Lys 11, rather than the 2.65 to 2.75 Å for the lysine or arginine P1 residues in complexes with hirulog-1 (PDB 1HGT), the substrate-like inhibitor avathrin, or an uncleavable fibrinogen substrate mimetic (PDB 1IHS) (19, 21, 22). Interference with nucleophilic attack of ^T^Ser 195 on the P1 carbonyl carbon by water hydrogen bonded with ^T^Ser 195 and the suboptimal distance between the serine hydroxyl and the carbonyl carbon of ^X^Lys 11 may help to explain the lack of hydrolysis of XC-43 by thrombin while other substrate-like inhibitors such as variegin (PDB 3B23), avathrin, and hirulog-1 are readily cleaved (19, 22, 23).

C-terminal to ^X^Lys 11, the path of the peptide continues along the thrombin surface toward exosite I. As discussed above, ^X^Leu 12 (P1ʹ) occupies the S1ʹ pocket bounded by ^T^Lys 60F, ^T^Trp 60D, ^T^His 57 and ^T^Cys 42. In this position, it is superimposable with the side chain ^V^His 12, the P2ʹ residue of variegin as modeled by Koh *et al.* (PDB 3B23, (23)). The P2ʹ residue of XC-43, ^X^Tyr 13, is inserted into the S2ʹ pocket at the base of the autolysis loop where its phenolic hydroxyl is hydrogen bonded with ^T^Asn 143. Its side chain is also covered by the peptide main chain in the vicinity of ^X^Glu 17 resulting in apparent exclusion from bulk solvent. ^X^Gln 14 interacts *via* a stacking arrangement with the side chain of ^T^Trp 60D, which is contained in a pocket formed by ^X^Gln 9 and ^X^Gln 14 from the inhibitor and ^T^Tyr 60A from thrombin (Fig. 4*D*). The carbonyl oxygen of XGln 14 also forms a hydrogen bond with the amide nitrogen of ^T^Asp 223 of an adjacent thrombin molecule. The main chain of the inhibitor forms a turn in this area and loses contact with the thrombin surface until ^X^Glu 17, whose carbonyl oxygen forms a hydrogen bond with the side chain of ^T^Gln 151. Notably, the side chain of ^X^Arg 15 interacts electrostatically with the side chain of ^T^Glu 146 of an adjacent thrombin molecule. The side chain of ^X^Glu 17 also makes extensive contact with the aromatic ring of ^X^Tyr 13, packing it against the thrombin surface, while the carbonyl oxygen from ^X^Gly 18 forms a hydrogen bond with the amide nitrogen of thrombin ^T^Leu 40, the carbonyl of ^X^Asn 20 forms a hydrogen bond with the side chain of ^T^Arg 73 and the backbone amide of ^X^Glu 23 hydrogen bonds with the carbonyl of ^T^Thr 74 as the chain continues to the area of exosite I (Fig. 4*E*).

At exosite I, XC-43 traverses the contact surface described for the structure of thrombin complexed with the central E region of fibrinogen (24). The peptide structure in this region is quite similar to that of other inhibitor complexes including those of hirudin and avathrin, which have similar α-helical structures at their C-termini, but specific side chain interactions are not highly conserved. Exosite binding is mediated largely by nonelectrostatic interactions involving hydrophobic residues including ^X^Ile 25, ^X^Val 29, and ^X^Leu 30. Additionally, the amide nitrogen of ^X^Ile 25 is hydrogen bonded to the side chain of ^T^Gln 38 of thrombin (Fig. 4*F*).

### XC-43 interferes with coagulation *in vitro*, *ex vivo*, and *in vivo*

**Figure 5:**
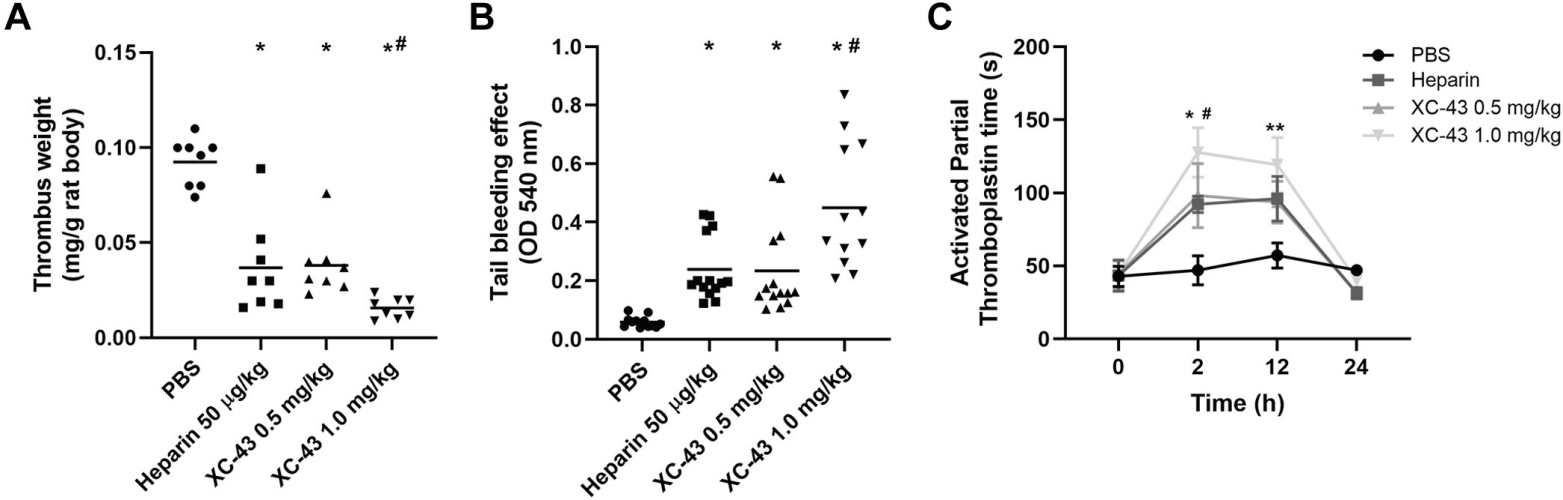
**XC-43 interferes with coagulation *in vitro* and *in vivo*.***A*, effect of XC-43 treatment (0.5 and 1.0 mg/kg) on thrombus formation induced by thromboplastin in rats. ANOVA analysis shows the presence of statistically significant difference between all treated groups with PBS (∗) and between XC-43 (1 mg/kg) *versus* Heparin (#). *B*, bleeding from the tail of rats after treatment of XC-43 (0.5 and 1.0 mg/kg). Heparin and XC-43-treated animals presented significant statistical difference when compared with the PBS group (∗). Difference was also observed between heparin and XC-43 (1 mg/kg) groups (#). *C*, *ex vivo* activated partial thromboplastin time (aPTT) of plasma from rats treated with XC-43 (0.5 and 1.0 mg/kg). For all *in vivo* experiments, heparin (50 μg/kg) was used as a control for anticoagulation. ANOVA analysis shows significant statistical difference at 2 h between PBS *versus* XC-43 (0.5 mg/kg) (∗) and PBS *versus* XC-43 (1 mg/kg) (#). Statistical difference was also found at 12 h between PBS and XC-43 (1 mg/kg) (∗∗).

Since XC-43 is a specific thrombin inhibitor, we evaluated its anticoagulant activity *in vitro* and *in vivo*. Addition of XC-43 to human plasma increased the prothrombin time (PT) and activated partial thromboplastin time (aPTT) by 2.3- and 3.5-fold, respectively, at a concentration of 0.4 μM and prolonged the thrombin time (TT) more than tenfold at a concentration of 0.2 μM (Table 2). Using rat models of thrombosis, we observed that XC-43 can interfere with coagulation *in vivo*. Intraperitoneal injection of XC-43 (0.5 mg/kg or 1 mg/kg) resulted in a reduction of calcium thromboplastin-induced thrombus weight by 58.2% and 83% respectively, in comparison to the PBS-injected group (Fig. 5*A*). Notably, XC-43 (1 mg/kg) was able to reduce thrombus weight by 57.3% in relation to animal treated with heparin (50 μg/kg) (Fig. 5*A*). The effect of XC-43 on coagulation was also evaluated using a tail bleeding assay, in which intraperitoneal injection of XC-43 (0.5 mg/kg or 1 mg/kg) resulted in an increased bleeding by 3.2 and 7.6-fold, respectively, when compared with PBS-injected animals, and 2.3-fold (XC-43, 1 mg/kg) when compared with heparin-treated animals (Fig. 5*B*). The aPTT was evaluated *ex vivo* at 0, 2, 12, and 24 h posttreatment. Animals treated with XC-43 (0.5 mg/kg) presented similar results as animals treated with heparin (50 μg/kg), in which an increase of twofold was observed at 2 and 12 h in relation to PBS-treated animals, while a threefold increase was found in animals treated with higher concentration of XC-43 (1 mg/kg) (Fig. 5*C*). No differences between treated and control groups were observed at 0 and 24 h.

**Table 2:** *In vitro* effect of XC-43 on coagulation

XC-43 (μM)	PT	aPTT	TT
0	13.6 s	34.3 s	34.4 s
0.2	21 s	98.4 s	>300 s
0.4	31.1 s	121.4 s	>300 s
0.8	>300 s	>300 s	>300 s

## Discussion

Anticoagulants from blood-feeding animals generally target either factor Xa or thrombin, reflecting the importance of these enzymes in both the intrinsic and extrinsic pathways of coagulation. Common among arthropod-derived thrombin inhibitors are proteins and peptides that block the active site of the enzyme and the fibrinogen-binding exosite I. Inhibitors from ticks (18), mosquitoes (15), and triatomines (25) have been shown to interact with thrombin at these two sites but unlike XC-42 and XC-43, some are large polypeptide chains with one or two domains. Notable exceptions to the catalytic site/exosite I mechanism do exist and include triabin from the triatomine bug *Triatoma pallidipennis*, which is a lipocalin-like protein that binds only to exosite I, leaving the catalytic site free (26) haemadin from the leech *Haemadipsa silvestris* (27), TTI from the tsetse fly *Glossina morsitans* (28), madanin from the tick *Haemaphysalis longicornis* (14, 17), and the hyalomins from *Hyalomma marginatum* (18, 29), which bind at the active site of thrombin, but also interact with the heparin-binding site (exosite II) rather than exosite I. XC-42 and XC-43 are compact catalytic site/exosite I inhibitors that are best compared structurally with hirudin from the medicinal leech, *H. medicinalis* (30), variegin from the tick *A. variegatum*, and avathrin also from *A. variegatum*. All of these peptides contain a characteristic C-terminal motif E-x-I-P-x(0,1)-[ED]-x-[L] that facilitates interaction with exosite I. Anophelin and cE5, from *Anopheles* mosquitoes, differ from the tick peptides, hirudin and XC-42/43, in that they bind exosite I at their N-terminal segments and block the catalytic site at their C-termini, running along the thrombin surface in a direction opposite to the other peptides (15, 31). Hirudin interacts with exosite I similarly to variegin and avathrin but blocks access to the catalytic site with its N-terminus rather than binding in a substrate-like manner as do the tick- and flea-derived peptides.

In the crystal structure of the XC-43-thrombin complex, the substrate-like arrangement of the inhibitor at the thrombin active site, with ^X^Lys 11 occupying the P1 position, suggests that the peptide would be cleaved, but surprisingly is not. In solution, XC-42 and XC-43 are cleaved to a relatively small degree when incubated at 37° for 2 h at a molar ratio of 25:1 (inhibitor:thrombin), indicating that they are somehow resistant to cleavage by α-thrombin. The tick peptides variegin, madanin, and hyalomin show complete cleavage when incubated with thrombin in solution at molar ratios of 25 to 30:1 (inhibitor:thrombin) at 37 °C for 2 to 3 h (16, 18, 20). Variegin has also been shown to be substantially cleaved after 30 min of incubation under these conditions (16). In published crystal structures, variegin, avathrin, and madanin as well as the synthetic peptide hirulog-1 are bound with thrombin but appear fully cleaved and at least partially dissociated from the enzyme (17, 19, 22, 23). Conversely, XC-43 appears well ordered in the binding groove, with the ^X^Lys 11-^X^Leu 12 peptide bond showing no evidence of even partial cleavage at a measured pH of the ∼7 in the crystallization solution. In structures of avathrin, variegin, and hirulog-1 complexed with thrombin, the residues immediately C-terminal to the scissile bond are either not visible or exhibit high levels of disorder, while the P1ʹ and P2ʹ residues of XC-43 appear to fully occupy their respective binding subsites (19, 22, 23).

In the XC-43-thrombin structure, the side chain of ^T^Ser 195 is turned approximately 180° from its position in complexes of thrombin with the inhibitors hirulog-1 and avathrin. It is also hydrogen bonded to a water molecule contained in a pocket formed in part by the side chain of the P1ʹ residue ^X^Leu 12. The hydrogen bonded water may disrupt the activation of ^T^Ser 195 in the catalytic triad, making it less nucleophilic. The change in position of the hydroxyl group moves it ∼3.6 Å from the carbonyl carbon atom of ^X^Lys 11 rather than ∼2.7 Å in the hirulog and avathrin structures. The structure of the PPACK adduct (PDB 1PPB (32)) with thrombin resembles a reaction intermediate in thrombin cleavage and shows ^T^Ser 195 to be oriented like the avathrin complex and not like XC-43, suggesting, along with other stable serine protease-inhibitor complexes such as BPTI with trypsin (PDB 3FP6 (33)) or rhodniin with thrombin (PDB 1TBR (25)), that this is the optimal orientation for reaction.

In the structure of the variegin–thrombin complex, it has been noted that after cleavage the positioning of ^V^His 12 (P2ʹ) appears to disrupt the charge relay system of thrombin by moving ^T^Ser 195 and ^T^His 57 further apart (23), while in the avathrin complexes, the relay system appears to be intact, showing nearly ideal hydrogen bonding distances between the residues of the catalytic triad. In the XC-43-thrombin structure, ^T^Ser 195 is oriented similarly to the variegin complex (19) but remains within hydrogen bonding distance of ^T^His 57. Since the residues directly C-terminal to the scissile bond are either disordered or change positions in the cleaved variegin and avathrin structures, it may be difficult to determine how they might be oriented prior to cleavage and whether differences in amino acid identity at the P1ʹ and P2ʹ positions might change susceptibility to cleavage.

XC-42 and XC-43 contain large hydrophobic or aromatic side chains and the P1ʹ and P2ʹ positions that may play a role in their enhanced stability. ^X^Leu 12 displaces ^T^Lys 60F from its normal site in the S1ʹ pocket much like peptidomimetic thrombin inhibitors having a having a benzothiazole group designed specifically to target the S1ʹ subsite (34). Slow dissociation of the P1ʹ residue could favor reformation of the peptide bond by maintaining proximity of the free amino group to acylated ^T^Ser 195 and preventing entry of a bound water molecule required for hydrolysis of the acyl enzyme (33). The backbone conformation of the peptide differs considerably from variegin over the residue range from P1ʹ to P5ʹ and the two complexes are not strictly comparable over this range. ^X^Tyr 13 at the P2ʹ position of XC-42 and XC-43 is not conserved in variegin and avathrin and the burial of its side chain by a combination of thrombin and inhibitor residues may provide additional stability to the complex that is lacking in other inhibitor complexes. It is true that unlike XC-43, Laskowski inhibitors are made conformationally rigid by networks of hydrogen bonds and disulfide linkages (35). Solution reaction data presented here show that while XC-43 is more stable than other similar inhibitors, it is cleaved more readily than highly stable Laskowski inhibitors such as BPTI (36). In the crystal, contact with adjacent molecules could also provide extra stabilization in slowing product release relative to the solution phase. ^X^Gln 14 and ^X^Arg 15 contact an adjacent thrombin molecule forming three intermolecular electrostatic interactions, but these are rather distant from the scissile bond.

At exosite I, the backbone position of XC-43 is similar to that found in the hirudin, avathrin, and variegin–thrombin complexes. However, despite the presence of the negatively charged E-x-I-P-x(0,1)-[ED]-x-[L] motif, the interactions between XC-43 and thrombin exosite I are mainly hydrophobic rather than electrostatic, while in the structures of hirudin and avathrin complexed with thrombin, mixed hydrophobic and electrostatic interactions are seen (19, 30). In hirudin, a tyrosine residue contained in this sequence is sulfated, a modification that significantly enhances the affinity of the peptide for thrombin, but in XC-43, the corresponding residue is replaced by valine (^X^Val 29, Fig. 1) indicating that tyrosine sulfation in the exosite I-binding region does not occur. Since the organisms producing anticoagulant peptides containing the charged exosite I-binding motif are phylogenetically distant from one another and have evolved the habit of blood feeding independently, any sequence similarity in this region can be attributed to convergent evolution. One possible explanation for conservation of the exosite-binding motif, despite the differences observed in specific amino acid interactions of stable thrombin-inhibitor complexes, would be that complementary charged sequences in the inhibitor and thrombin exosite I facilitate the initial association of the two molecules (*i.e.*, electrostatic steering) and further orientation of the inhibitor in order to form a tightly bound complex (*i.e.*, ionic tethering) (37, 38).

The biochemical and structural features of XC-43 suggest that it would show excellent anticoagulant activity *in vivo*. This proved to be the case as tail bleeding and venous thrombosis assays demonstrated that animals treated with XC-43 (0.5 mg/kg) presented similar results to those treated with heparin (50 μg/kg). XC-43 at 0.5 mg/kg reached a maximum theoretical blood concentration of 2.0 μM, assuming no losses and a weight of 250 g/animal (16 ml blood volume). Inhibition of thrombosis by XC-43 therefore occurs at lower concentrations than observed with thrombin inhibitors described from other blood feeding arthropods and the synthetic analog hirulog (16, 18, 19). Furthermore, unlike hyalomin (from the tick *H. marginatum rufipes*), madanin (from the tick *H. longicornis*), avathrin, and variegin, XC-43 is not cleaved by thrombin, suggesting that it retains full inhibitory activity and affinity for thrombin after interaction with the enzyme. Recently Agten *et al.* (39) developed synthetic inhibitors by fusing the exosite II-binding region of the tsetse thrombin inhibitor (TTI) (40) with variegin or anophelin generating a molecule with high affinity for thrombin that binds both exosites in addition to the catalytic site. A similar strategy using XC-43 instead of variegin would be interesting to pursue, since the flea-derived peptide is not cleaved by thrombin and therefore could result in a more stable complex. Taken altogether, the XC-43-thrombin complex presented here provides new insights into the interactions that take place at the thrombin prime sites and how they can contribute to the stability of the complex that could be incorporate in the design of new compounds.

It seems certain that thrombin is the main target of flea saliva in the coagulation cascade and that XC-42 and XC-43 are the specific salivary inhibitors of this protease. This conclusion is based on the selectivity of SGH and XC-43 toward thrombin when evaluated alongside other proteases from the coagulation cascade, as well as the high affinity of the inhibitor for the protease determined in kinetic and SPR experiments (pM range). Both *X. cheopis* SGH and XC-43 inhibit thrombin function in a concentration-dependent manner and prolong the PT, aPTT, and TT *in vitro*, indicating specific inhibition of thrombin that is capable of significantly delaying the formation of a fibrin clot in whole plasma. The N-terminal insertion seen in XC-42 lies outside of the region interacting with thrombin and appears not to have a significant effect on its function. The two inhibitors are apparently functionally equivalent. At this point, only limited functional analysis of *X. cheopis* saliva has been performed, but transcriptomic studies have shown expansion of gene families encoding apparently catalytically inactive phosphatases as well as scorpion toxin-like proteins that may modulate host physiological systems related to blood feeding including platelet responses, inflammation, and pain. XC-42 and 43 are the first flea salivary components identified as targeting the hemostatic system. Their potency and specificity suggest that flea saliva will be a rich source of potentially useful physiological mediators.

## Experimental procedures

### Flea salivary gland dissection

SGH was prepared using intact salivary gland pairs collected from adult female *X. cheopis* fleas as previously described (12). Pools of 1000 pairs of glands were disrupted in 1 ml of phosphate-buffered saline (PBS) pH 7.4 and centrifuged for 10 min, 12,000*g* at 4 °C. The supernatant was collected, and the total protein concentration determined with the BCA Protein Kit assay (Thermo Fisher Scientific).

### Mass spectrometry analysis

Approximately 11 μg of salivary gland homogenate was reduced in 50 μl of 50 mM HEPES, 5 mM DTT, pH 8.0 at 37 °C for 40 min. The solution was cooled to room temperature and iodoacetate was added and the mixture incubated for 20 min. The protein mixture was digested with trypsin (600 ng) for 15 h at 37 °C. The reaction was performed in 0.5% trifluoroacetic acid (TFA), and the peptides were then desalted and concentrated using an Optimize micro scale polymer cartridge. Peptides were eluted with 100 μl 50% acetonitrile, 0.1% TFA, and dried under vacuum at 50 °C. Finally, the mixture was dissolved in injection solvent (0.1% formic acid, 3% AcCN), the peptides quantified fluorometrically and adjusted to a final concentration of 200 ng/μl with injection solvent.

The LC-MS experiment was performed using Orbitrap Fusion Lumos mass spectrometer (Thermo Fisher Scientific) coupled to EASY nLC 1200 nano-liquid chromatography system (Thermo Fisher Scientific). Peptides were first bound to a PepMap C18 column (3 μm particle, 100 Å pore, 75 μm inner diameter, 2 cm length), then separated using an EASY-Spray analytical column (PepMap C18, 2 μm particle, 100 Å pore, 75 μm inner diameter, 25 cm length) using a linear gradient of 0 to 40% acetonitrile in water containing 0.1% formic acid for 80 min, (followed by 40–80% for 5 min, 80% hold for 5 min, 80–50% for 5 min, 0% hold for 5 min). The analytical column was kept at 50 °C. Data acquisition was done with the standard data-dependent acquisition strategy, where the survey MS1 scan was done at least every 2 s with Orbitrap mass analyzer at 120,000 resolution and the MS2 scans were done with a linear ion trap mass analyzer for multiply charged precursor ions isolated with the 1.6 m/z window using a quadrupole and fragmented by CID at 35% collision energy. The EASY-IC internal calibration was utilized for Orbitrap scans, and the dynamic exclusion period was set at 15 s. Tandem mass spectra were extracted from Thermo RAW files using RawExtract 1.9.9.2 (41) and analyzed using the PatternLab for proteomics 4.0 platform (42) against a specific flea database. The database was created by combining all the deposited sequences at NCBI from *C. felis* (until 11/18/2019), the sequences reported in the salivary gland transcriptome of *X. cheopis* (12) (36,610 entries) and reverse sequences of all entries. The search space included all fully tryptic and half-tryptic peptide candidates. Carbamidomethylation of cysteine was used as a static modification. Data was searched with a 50 ppm precursor ion tolerance, a 0.4 Da fragment ion tolerance, and with a number of missed cleavages of 2. The validity of the peptide spectrum matches (PSMs) generated by Comet was assessed using the Search Engine Processor (SEPro) module from PatternLab for Proteomics 4.0 platform (42). A cutoff score was established to accept a protein false discovery rate (FDR) of 1% based on the number of decoys. Results were postprocessed to only accept PSMs with <10 ppm precursor mass error and proteins with a unique peptide. Normalized spectral abundance factors (NSAFs) were used to represent relative abundance.

To detect cleavage of XC-42 and XC-43 by thrombin in solution, we incubated each peptide separately at a concentration of 25 μM in the presence of thrombin (1 μM) in 25 mM Tris-HCl pH 8.0 containing 150 mM NaCl for 2 h at 37 °C. Reactions were stopped by adjusting the pH to 2.0 with the addition of TFA. A Q Exactive plus (Thermo Fisher Scientific) mass spectrometer was used to carry out ESI-MS experiments. The instrument was operated at a resolution of 280k, spray voltage of 3.5 kV, and capillary temperature of 250 °C. Samples were desalted by C18 Zip-tip (Millipore) and dissolved in 200 μl of reconstitution buffer (50% acetonitrile, 49% water, 1% TFA). Samples were introduced using a syringe pump (Thermo Fisher Scientific) with a flow rate of 5 μl/min. Xtract was used to deconvolute the raw data.

### Peptide synthesis

XC-42 and XC-43 peptides (12) were synthesized by Peptide 2.0 Inc, analyzed for quality, and purified to approximately 98.6%.

### Blood clotting assays

Partial thromboplastin time (aPTT), PT, and TT were evaluated on a STart 4 coagulometer (Diagnostica Stago). For the aPTT and PT assays, freeze-dried, citrated, normal human plasma was resuspended in ultrapure water. For the aPTT assay, plasma (50 μl) and 50 μl of prewarmed aPTT reagent 9 (STA PTT; Diagnostica Stago) were incubated with 5 μl XC-43 (0.2–3.2 μM) or Tris-buffered saline, pH 7.4 (TBS) (control) and placed in the coagulometer for 5 min at 37 °C. Calcium chloride (50 μl at 25 mM) was added to start reactions. For the PT, plasma (50 μl) was incubated with 5 μl XC-43 (0.2–3.2 μM) or TBS (control) and placed in the coagulometer for 5 min at 37 °C. Then, 100 μl of the PT reagent (NEOplastine CI plus; Diagnostica Stago) was added. For the TT test, thrombin (40 nM) was incubated with 5 μl XC-43 (0.2–3.2 μM) or TBS (control) in a 50 μl total volume and placed in the coagulometer for 5 min at 37 °C. Then, 100 μl of human fibrinogen (5 mg/ml) was added. Time for clot formation was recorded in duplicate in two independent replicates.

### Protease inhibition assays

Human thrombin (2 nM), human factor XIa (15 nM), human factor XIIa (15 nM), human kallikrein (HuKLK) (0.5 nM), uPA (10 nM), human plasmin (10 nM), and human cathepsin G (Cat. G) (100 nM) were obtained from Enzyme Research Laboratories. Neutrophil elastase (HNE) (10 nM) and tPA (50 nM) were obtained from Molecular Innovations. Human chymase (10 nM) was obtained from Sigma Aldrich and bovine factor Xa (2 nM) from Hematologic Technologies. Substrates were used at 200 μM final concentration: H-D-Phe-L-Pip-Arg-pNA for thrombin; Bz-Ile- Glu(γ-OR)-Gly-Arg-pNA for factor Xa; H-D-Pro- Phe-Arg-pNA for factor XIa and factor XIIa; H-D-Val-Leu-Lys-pNA for plasmin; H-D-Pro-Phe-Arg-pNA for kallikrein; MeOSuc-Ala-Ala-Pro-Val-pNA for HNE, H-D-Ile-Pro-Arg-pNA for tPA and Glu-Gly-Arg-pNA for uPA (Diapharma Inc); N-Succinyl-Ala-Ala-Pro-Phe-pNA for cathepsin G and chymase (Sigma Aldrich). All assays were performed at 30 °C in triplicate using 96-well plates. XC-42 (1 μM), XC-43 (1 μM), or SGH (1 μg) was preincubated with each protease for 5 min in 20 mM Tris-HCl, 150 mM NaCl, 0.01% Tween-20, pH 7.4. After incubation, the corresponding substrate for each protease was added in a 100 μl final reaction volume. The substrate hydrolysis rate was followed at 405 nm in kinetic mode in a Thermo max micro plate reader (Molecular Devices). The observed substrate hydrolysis rate in the absence of XC-43 or salivary gland extract was considered as 100% and compared with the remaining enzymatic activity in the presence of the inhibitor. Data are presented as mean ± standard error of triplicate readings.

### Kinetic studies

All reactions were performed at 30 °C. Kinetic assays were performed using human α-thrombin (Enzyme Research Laboratories) and chromogenic substrate S-2238 (Diapharma) using a Thermomax micro plate reader (Molecular Devices). Reactions were performed in 100 μl of TBS buffer (10 mM Tris, 0.15 M NaCl, pH 7.4) containing 0.01% Tween-20. XC-43 (0–3.2 nM) or 0 to 15 pairs of *X.cheopis* salivary glands were incubated with human α-thrombin (0.2 nM) at 30 °C for 5 min followed by addition of S-2238 (50, 100, 200, 400, and 600 μM). Reactions were followed for 30 min. The inhibitory constants were determined by fitting the nonlinear regression model according to the Morrison’s equation (43) using the GraphPad Prism software (GraphPad Software, Inc).

### SPR assays

Evaluation of binding kinetics by SPR was performed using a Biacore T200 instrument. Synthetic XC-43 containing covalently linked biotin at the N-terminal amino group and the ε-amino group of Lys 1 was bound to a surface of immobilized neutravidin on a CM-5 chip. After conditioning, human α-thrombin was passed over the surface, and kinetic data were collected in single cycle mode using a running buffer of 10 mM HEPES pH 7.4, 150 mM NaCl (HBS). The data were fit to a 1:1 Langmuir binding model. Binding was also analyzed after immobilization of α-thrombin on a CM-5 chip using amine coupling methodology. XC-42 and XC-43 were passed over the surface, and data were collected in the single cycle mode using HBS as a running buffer. The data were fit to a 1:1 binding model as described above.

### Isothermal titration calorimetry

Isothermal titration calorimetric experiments were performed with a Microcal VP-ITC instrument at 30 °C. Human α-thrombin and XC-43 were dissolved in PBS, pH 7.4 at concentrations of 1 μM and 10 μM, respectively. XC-43 was added as 10-μl injections to the protein sample contained in the calorimeter cell. Calculated injection enthalpies were fit to a single-site binding model in the Microcal data evaluation software.

### Thrombin-induced platelet aggregation

Platelet-rich plasma (PRP), from healthy donors having given informed consent (NIH/CC/DTM), was centrifuged at 1100*g* for 15 min at room temperature and the pellet resuspended in same initial volume of Tyrode buffer (5 mM HEPES pH 7.4, 137 mM NaCl, 2 mM KCl, 1 mM MgCl_2_, 12 mM NaHCO_3_, 0.3 mM NaH_2_PO_4_, 5.5 mM glucose, 1.5 mg/ml BSA). Aggregation was monitored at 37 °C in an aggregometer (Lumi-aggregometer, Chrono-log Corporation) by incubating 100 μl of washed platelets with XC-43 (0–30 nM) or SGH (3.0 μg) in 200 μl of Tyrode buffer for 1 min at 37 °C. Aggregation was initiated by adding human α-thrombin (3 nM). Each measurement was performed in triplicate.

### XC43-thrombin complex crystallization

Prothrombin (Enzyme Research Laboratories) activation was performed in 20 mM Tris, 0.1 M NaCl, 1 mM EGTA, 10 mM CaCl_2_ pH 7.5, and 1% of *Oxyuranus scutellatus* venom at 37 °C for 40 min. Activated thrombin (α-thrombin) was purified using a HiTrap heparin column pre-equilibrated with 20 mM Tris, 0.1 M NaCl, 1 mM EGTA, pH 7.5. Proteins were eluted with 20 mM Tris, 1 M NaCl, 1 mM EGTA pH 7.5 and concentrated in 10 mM HEPES pH 7.4 using a 10 kDa Amicon (Merck). The XC-43-thrombin complex was assembled in 10 mM HEPES pH 7.4 with a 1:1.5 M ratio (thrombin: XC-43) at room temperature for 1 h. The complex was concentrated to 11.4 mg/ml and crystallized using the hanging drop vapor diffusion method in 0.2 M magnesium acetate, 9% PEG 8000 (measured pH ∼ 7) at room temperature. After growth, crystals were flash cooled in liquid nitrogen in 0.2 M magnesium acetate, 12% PEG 8000, 15% glycerol.

### X-ray diffraction data collection and structure solution

Diffraction data were collected at beamline 22-ID of the Southeast Regional Collaborative Access Team (SER CAT) at the Advanced Photon Source (Argonne National Laboratory) and processed using XDS (44). The complex crystallized in the space group P2_1_2_1_2_1_ with six complexes contained in the asymmetric unit (Table 1). The structure was solved by molecular replacement with Phaser using a deposited thrombin structure (PDB accession 1PPB (32)) with the ligand removed as a search model. The XC-43 model was built manually using Coot (45), and the complex was refined using phenix.refine (46) with a TLS model applied (Table 1).

### *In vivo* experiments

#### Animals

The *in vivo* experiments were carried out in the Experimental Research Center of Hospital de Clínicas de Porto Alegre (HCPA). Male Wistar rats (weighing 250–300 g) were housed in a temperature-controlled room (21–25 °C, in a 12-h light/dark cycle), with free access to water and food. All animal experiments followed the current legislation in Brazil, Law 11.794 (08/10/2008). The procedures were based on the Brazilian Guideline for the Care and Use of Animals for Scientific and Educational Purposes–DBCA (RN 30/2016) and on the National Institutes of Health guide for the care and use of Laboratory animals (NIH Publications No. 8023, revised 1978). The euthanasia followed the Guidelines for Euthanasia Practice (2013) indicated by the CONCEA (National Council for Control of Animal Experimentation). All procedures performed in this study were in accordance with the ethical standards of Animal Use Ethics Committee–Hospital de Clínicas de Porto Alegre, and the study was approved by the Committee with the number 19-0497.

#### Studies on blood coagulation parameters

A total number of 24 animals were randomly distributed into four groups (n= 6 per group) and injected intraperitoneally (300 μl) with: (i) PBS; (ii) heparin (50 μg/kg); (iii) XC-43 (0.5 mg/kg); or (iv) XC-43 (1.0 mg/kg). Animals were anesthetized with isoflurane (5% for induction; 2% for maintenance) and blood samples were obtained at 0, 2, 12, and 24 h posttreatment in 1:10 (v/v) 3.8% trisodium citrate. The PPP was obtained by blood centrifugation (1500*g* for 10 min) and the aPTT evaluated using the APTT ellagic kit (Wiener Lab). Clot formation was monitored at 650 nm using a SpectraMax M3 (Molecular Devices).

#### Tail bleeding assay

The tail bleeding assay was performed with 24 animals kept at 37 °C under general anesthesia with isoflurane vaporized in 100% oxygen at a dose of 5% for induction and 2% for maintenance (flow rate of 0.5 l/min). These animals were randomly distributed into four groups (n = 6 per group) and injected intraperitoneally (300 μl) with: (i) PBS; (ii) heparin (50 μg/kg); (iii) XC-43 (0.5 mg/kg); or (iv) XC-43 (1 mg/kg). After 30 min posttreatment, a medium depth incision was performed at 3 mm from the tip of the animals' tail; the tail was submerged in a test tube containing saline solution (4 ml) and maintained for 30 min. Then, samples from saline solution were appropriately diluted and the absorbance at 540 nm was determined spectrophotometrically.

#### Deep vein thrombosis

Wistar rats (total number of 32) were randomly distributed into four groups (n = 8/group) injected intraperitoneally (300 μl) with: (i) PBS; (ii) heparin (50 μg/kg); (iii) XC-43 (0.5 mg/kg); or (iv) XC-43 (1 mg/kg). After 30 min, the animals were anesthetized with isoflurane as described above and maintained at 37 °C in a thermal surgical table. Then, a laparotomy was performed, and the caudal vena cava was carefully dissected from surrounding tissues. Venous thrombosis was induced by calcium thromboplastin (3 mg/kg) injection directly into the vena cava (near to the right renal vein) and stasis was immediately established by the ligation of caudal vena cava (above the insertion point of the right renal vein). The distal ligations of the vena cava (above the common iliac veins confluence), left renal vein, and other major tributaries were conducted 20 min after thromboplastin injection. The isolated segment of the caudal vena cava was removed and carefully dissected to obtain the thrombus, which was rinsed in cold saline solution, dried on a filter paper at 60 °C (1 h), and weighed. The ratio of thrombus per rat weight was calculated and used for comparisons between groups.

#### Statistical analyses of *in vivo* data

Results are expressed as mean ± SEM. The significance of differences between mean values of two experimental groups was determined using Student's *t* test. When more than two groups were compared, an analysis of variance was used, followed by a Bonferroni's test to compare pairs of means. A *p* value of less than 0.05 was chosen to establish significance. Statistical analysis was performed using GraphPad Prism (GraphPad Software Inc).

## Data availability

Coordinates and structure factors for the XC-43-thrombin complex have been deposited in the wwPDB with the accession number 7MJ5. The mass spectrometry proteomics data have been deposited to the ProteomeXchange Consortium *via* the PRIDE (47) partner repository with the dataset identifier PXD028851.

## Supporting information

This article contains supporting information.

## Conflict of interest

The authors declare that they have no conflicts of interest with the contents of this article.
