# Supplementary material for: Identification of a substrate-like cleavage-resistant thrombin inhibitor from the saliva of the flea Xenopsylla cheopis
Source: J Biol Chem. 2021 Oct 21;297(5):101322. doi: 10.1016/j.jbc.2021.101322 (PMC8573170; doi:10.1016/j.jbc.2021.101322)
Supplement: Supplemental Figures S1–S6 and Table S1 [file mmc1.pdf]

Table S1: XC-42 and XC-43 identification by LC-MS.

Accession	Length	Unique peptides	Molecular Weight	Sequence Count	Spectrum Count	NSAF	Coverage	Protein Score
**ABM55431.1**	73	12	8008	31	434	0.061099	0.6575	1693.786
**ABM55432.1**	59	1	6505.3	20	70	0.012193	0.5424	219.9761

*NSAF: Normalized spectral abundance factors.

Fig. S1

```
          1        10        20         30        40        50
          |        |         |          |         |         |
XC-42 : KPVEAEVAQSNLDECEVEAEVAQPKLYQRGEGGNGMEPIPEDVLNEALNA
XC-43 : KP--------------VEAEVAQPKLYQRGEGGNGMEPIPEDVLNEALNA
```

Figure S1: Alignment of XC-42 and XC-43 showing the unique peptides (red) identified by LC-MS/MS. Residue numbers refer to the mature peptide.

Fig. S2

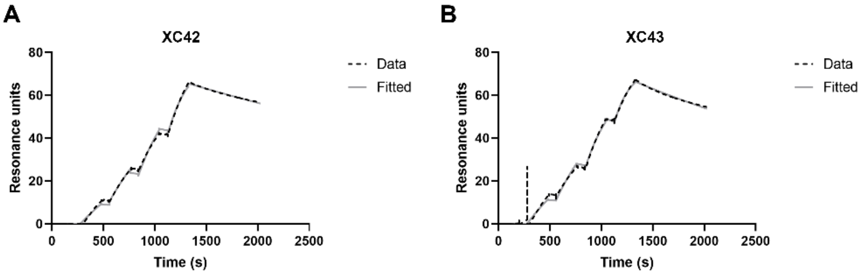

C

	XC42	XC43
ka (1/Ms)	9.84×10^{4}	1.31×10^{5}
kd (1/s)	1.953×10^{-4}	3.04×10^{-4}
K_D (M)	1.98×10^{-9}	2.32×10^{-9}

Figure S2: Surface plasmon resonance in the single cycle mode of **(A)** XC-42 or **(B)** XC-43 binding to thrombin immobilized on a CM5 chip surface. Experimental data are shown as a solid grey line while the calculated fit is shown as a dashed line **(C)** The kinetic parameters were estimated by fitting a 1:1 binding model. Peptide concentrations for each experiment were, 0 nM, 6.25 nM, 12.5 nM, 25 nM, 50 nM.

Fig. S3

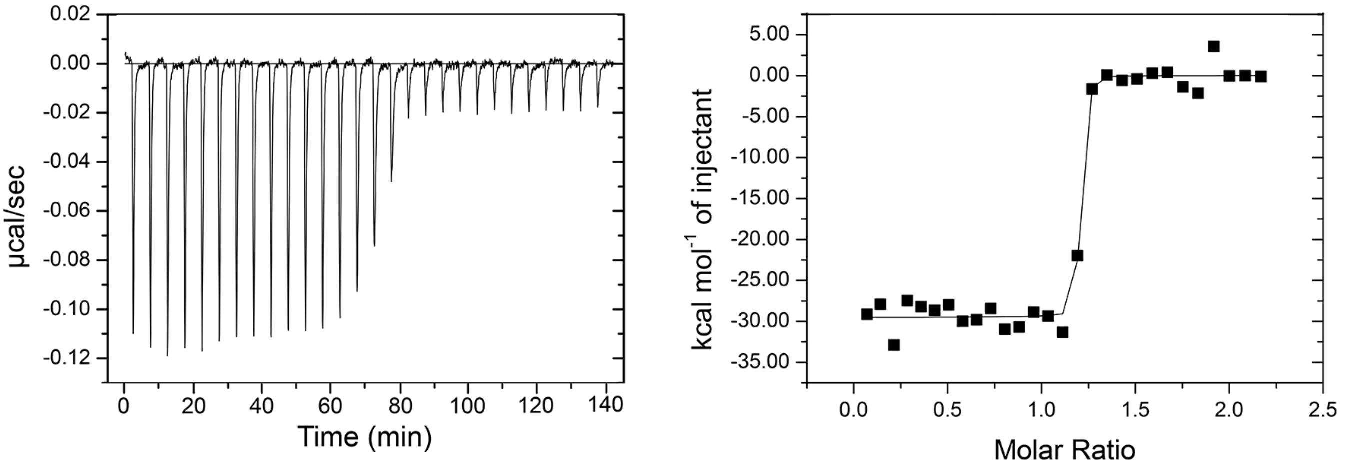

Figure S3: Analysis of XC-43 binding with thrombin using isothermal titration calorimetry. XC-43 (10 µM) was added to a solution of thrombin (1 µM) in a series of 10 µl injections. Heats were recorded on a VP-ITC MicroCalorimeter and the data fitted using a single binding site model. The high affinity makes the magnitude of the equilibrium constant impossible to extract from these data, but using a maximum "c" value (1) of 500 the association constant (K) must be $\geq 5 \times 10^8$ M^{-1} ($K_D \leq 2$ nM) with a single peptide binding site per thrombin molecule.

1. Broecker, J., Vargas, C., and Keller, S. (2011) Revisiting the optimal c value for isothermal titration calorimetry. *Anal Biochem* **418**, 307-309

Fig. S4

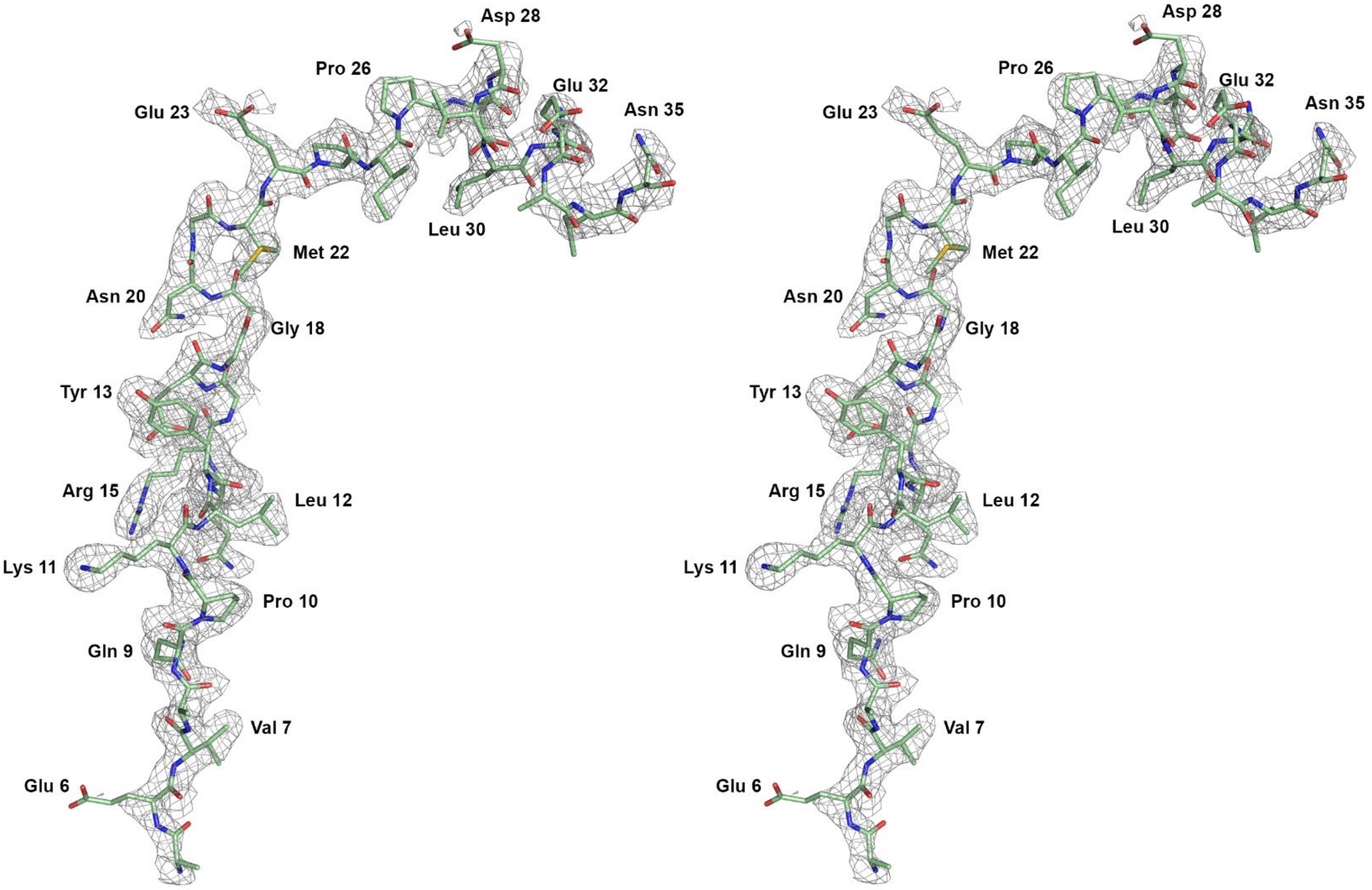

Figure S4: Stereo view of the final XC-43 model (chain Q) covered by Fo – Fc omit density contoured at 2.0 σ. The map was produced after simulated annealing (cartesian) using phenix.refine after removal of coordinates for all six XC-43 chains. Carbon atoms are shown in green; nitrogen atoms are represented in blue, oxygen atoms in red and sulfur atoms in yellow.

Fig. S5

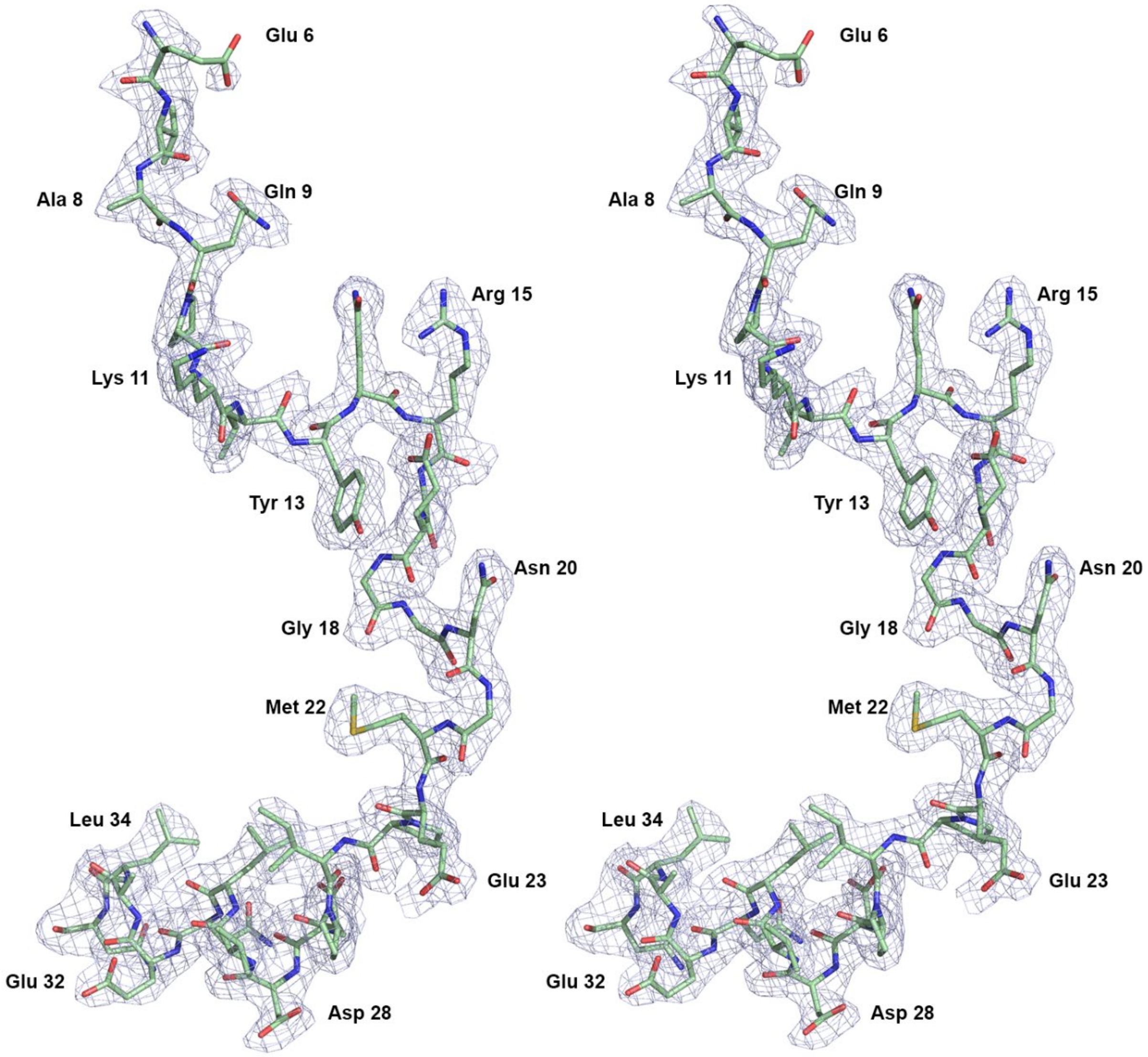

Figure S5: Stereo view of the final XC-43 model (chain Q) with 2Fo – Fc density contoured at 1.0 σ covering the ligand. Carbon atoms are shown in green; nitrogen atoms are represented in blue; oxygen atoms in red and sulfur atoms in yellow.

Fig. S6

A B

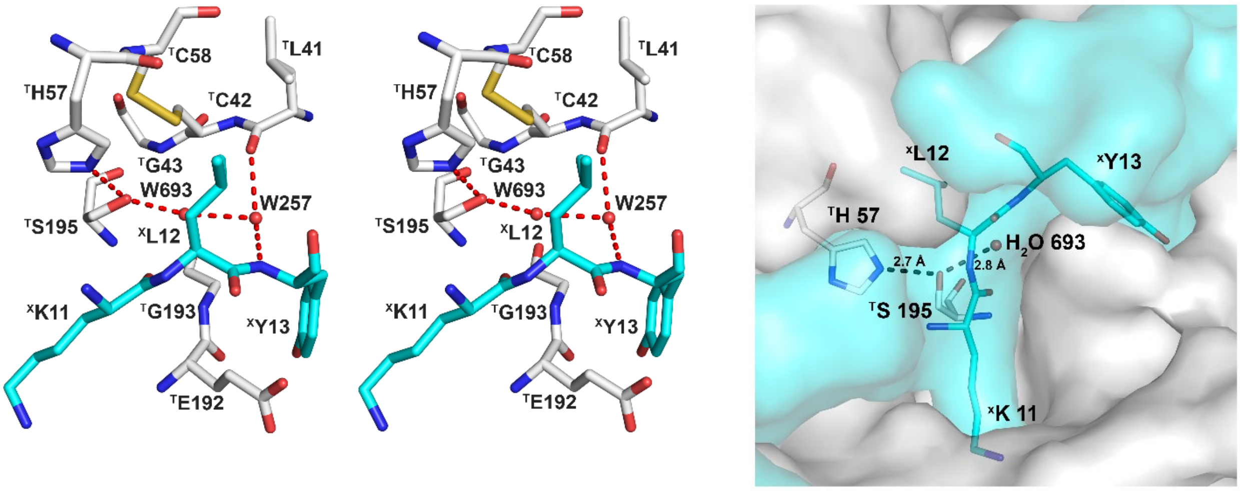

Figure S6: (A) Stereo representation of the trapped water molecule (red sphere) hydrogen bonded with the hydroxyl group of Ser 195. **(B)** The XC-43 surface is shown in cyan with Lys 11, Leu 12 and Tyr 13 represented as sticks with carbons atoms shown in cyan, nitrogen atoms in blue and oxygen atoms in red. Dashes represent hydrogen bonds with the distances (Å) between the designated atoms.
